# Comparison of the clinical and cost effectiveness of two management strategies (rehabilitation versus surgical reconstruction) for non-acute anterior cruciate ligament (ACL) injury: study protocol for the ACL SNNAP randomised controlled trial

**DOI:** 10.1186/s13063-020-04298-y

**Published:** 2020-05-14

**Authors:** Loretta Davies, Jonathan Cook, Jose Leal, Carlos Morgado Areia, Beverly Shirkey, William Jackson, Helen Campbell, Heidi Fletcher, Andrew Carr, Karen Barker, Sarah E. Lamb, Paul Monk, Sean O’Leary, Fares Haddad, Chris Wilson, Andrew Price, David Beard

**Affiliations:** 1 Nuffield Department of Orthopaedics, Rheumatology and Musculoskeletal Sciences, Botnar Research Centre, University of Oxford, Headington, Oxford, OX3 7LD UK; 2 Health Economics Research Centre, Nuffield Department of Population Health, University of Oxford, Department of Public Health, University of Oxford, Oxford, UK; 3 Nuffield Orthopaedic Centre, Oxford University Hospitals NHS Foundation Trust, Oxford, UK; 4 Royal Berkshire NHS Foundation Trust, Reading, UK; 5 University College Hospital, London, UK; 6 University Hospital of Wales, Cardiff, UK

**Keywords:** Anterior cruciate ligament deficiency, Randomised controlled trial, Reconstruction, Rehabilitation

## Abstract

**Background:**

Anterior cruciate ligament (ACL) rupture is a common knee injury that can lead to poor quality of life, decreased activity and increased risk of secondary osteoarthritis of the knee. Management of patients with a non-acute ACL injury can include a non-surgical (rehabilitation) or surgical (reconstruction) approach. However, insufficient evidence to guide treatment selection has led to high variation in treatment choice for patients with non-acute presentation of ACL injury.

The objective of the ACL SNNAP trial is to determine in patients with non-acute anterior cruciate ligament deficiency (ACLD) whether a strategy of non-surgical management (rehabilitation) (with option for later ACL reconstruction only if required) is more clinically effective and cost effective than a strategy of surgical management (reconstruction) without prior rehabilitation with all patients followed up at 18 months.

**Methods:**

The study is a pragmatic, multi-centre, superiority, randomised controlled trial with two-arm parallel groups and 1:1 allocation. Patients with a symptomatic non-acute ACL deficient knee will be randomised to either non-surgical management (rehabilitation) or surgical management (reconstruction). We aim to recruit 320 patients from approximately 30 secondary care orthopaedic units from across the United Kingdom. Randomisation will occur using a web-based randomisation system. Blinding of patients and clinicians to treatment allocation will not be possible because of the nature of the interventions. Participants will be followed up via self-reported questionnaires at 6, 12 and 18 months. The primary outcome is the Knee injury and Osteoarthritis Outcome Score (KOOS) at 18 months post randomisation. Secondary outcomes will include a return to sport/activity, intervention-related complications, patient satisfaction, expectations of activity, generic health quality of life, knee specific quality of life and resource usage.

**Discussion:**

At present, no evidence-based treatment of non-acute ACL deficiency exists, particularly in the NHS. Moreover, little consensus exists on the management approach for these patients. The proposed trial will address this gap in knowledge regarding the clinical and cost effectiveness of ACL treatment and inform future standards of care for this condition.

**Trial registration:**

ISRCTN: 10110685. Registered on 16 November 2016. ClinicalTrials.gov: NCT02980367. Registered in December 2016.

## Background and rationale

Anterior cruciate ligament (ACL) rupture is a common injury, mainly affecting young, active individuals with estimated 200,000 injuries annually in the United States [1]. ACL injury can have a profound effect on knee kinematics (knee movement and forces), with recurrent knee instability (giving way) as the main problem [2]. Furthermore, the injury can lead to poor quality of life, decreased activity [3] and increased risk of secondary osteoarthritis of the knee [4]. Some patients, once recovered from the initial injury, are able to function well without their ACL (copers), usually after undergoing some formal rehabilitation [5]. Other patients continue with episodes of knee instability, and surgery (ACL reconstruction using a graft) is thought necessary to stabilise the knee.

In the United Kingdom, a surgical management strategy has become the preferred treatment for individuals with ACL injuries. Our recent survey shows that the ratio of surgical intervention to non-surgical conservative intervention is 4:1 (unpublished data). Our data suggest that 80% of non-acute patients are now directly listed for surgery in the NHS. In England, an estimated 15,000 primary ACL reconstruction surgeries are performed each year [6]. However, this is a modest estimate based on Hospital Episode Statistics (HES) data, and the real figure for a UK population of 63 million may be closer to 50,000 pa (based on Swedish ACL registry data - incidence 71/100,000 pa) [7]. Based on the conservative estimate (*n* = 15,000), the costs of ACL reconstruction to the NHS in 2015 was approximately £63 million.

Despite ACL reconstruction being common, the current management for ACL injury is based on limited evidence [5, 8–10]. A Cochrane systematic review examined whether surgery or non-surgical (conservative) management was superior for ACL injury [11] and concluded no high-quality evidence exists on which to base practice. This uncertainty in the treatment of ACL patients is supported by the findings on the UK Database of Uncertainties about the Effects of Treatment NHS (DUETS). Surgical stabilisation of the knee joint appears a beneficial intervention, but whether the surgery is more beneficial than non-surgical intervention is unclear, particularly in the non-acute patient.

The unsupported preference for surgical management of the ACL deficient knee has recently been questioned further by evidence obtained in a Scandinavian trial [12]. The benefit of surgery, for all injured patients, was shown to be uncertain, with an operation being unnecessary in many cases. Frobell et al. [12] showed that a period of prior rehabilitation before considering operation can reduce ACL surgery by up to 50%. The clinical implication is that a period of rehabilitation should always be offered prior to surgical reconstruction, and this has become accepted practice, particularly with isolated ACL tears without comorbidity. However, whilst this clinical decision making evidence is valid for acutely injured individuals, it is not considered applicable to those more typically seen in the NHS, where patients are often non-acute, having sustained injury sometime earlier. By the time NHS patients are diagnosed and begin dedicated ACL injury management, up to 12 months can have passed since initial injury [13].

The mixed acknowledgement and uptake of this evidence and the uncertainty over the applicability to a less acute UK population has resulted in a highly varied approach to managing ACL injury in the NHS [14–16]. An overuse of surgical management may occur in the non-acute population, yet conversely, an argument may be made to bypass any formal rehabilitation and undergo immediate reconstructive surgery. Which strategy is the most clinically and cost effective remains unknown. Because surgery is expensive and may also have greater complications [6, 17], generating evidence for automatic default ACL reconstruction is even more important [18]. Likewise, the routine prescription of formal rehabilitation, if not beneficial, is considered wasteful and may disadvantage individuals with ACL injuries. The need exists to identify the most appropriate treatment strategy.

In terms of current research, a review of the Clinical Trials Registry found one other study examining the clinical and cost effectiveness of two treatment strategies for ACL rupture [19]. This trial is being carried out in the Netherlands and has a sample size of 188 participants. As it also evaluates the newly injured (acute) patients, this study replicates the Scandinavian study setting and, again, cannot be directly applied to the typical NHS pathway.

In summary, at present no evidence-base management of non-acute ACL deficiency is occurring, particularly in the NHS. Moreover, little consensus exists on the management of these patients. The proposed ACL SNNAP trial will address the gap in the evidence base regarding the clinical and cost effectiveness of these approaches and inform standards of care for ACL deficiency management in non-acute patients.

### Objectives

#### Primary objective

The primary objective of the ACL SNNAP study is to determine in patients with non-acute anterior cruciate ligament deficiency (ACLD) whether a strategy of non-surgical management (rehabilitation) (with option for later ACL reconstruction only if required) is more clinically effective and cost effective than a strategy of surgical management (reconstruction) without prior rehabilitation with all patients followed up at 18 months.

#### Secondary objectives

Secondary objectives are to compare the two management strategies regarding the return to activity/level of sports, generic quality of life, knee-specific patient-reported outcomes, intervention-related complications, health economics–cost effectiveness, ability to work (e.g., sickness absences/return to work number of days off work and subjective working ability), resource use and costs, expectations of return to activity and confidence in relation to the knee.

## Methods/Design

### Trial design

The ACL SNNAP trial is a pragmatic, multi-centre, superiority, randomised controlled trial with 2-arm parallel groups and 1:1 allocation ratio to compare non-surgical management (rehabilitation) or surgical management (reconstruction) options for patients with a symptomatic non-acute ACL deficiency. An internal pilot is included with clear progression criteria regarding recruitment.

### Internal pilot

A two-stage pilot study will be conducted to ensure recruitment and guarantee progression. For the first stage, the trial will not progress without the recruitment of a minimum of 25 patients (from eight centres) in the first 6 months of being open to recruit to the study. At this stage, a recruitment target for each centre will be at least one participant recruited or evidence provided through screening data of an active approach to recruitment. This will provide early evidence that centres are able to identify and recruit patients. A further review of progress will be made at 1 year from the start of recruitment, where 94 patients (from 12 to 18 sites) will meet the progression criteria, with a target set for each centre open to recruitment to achieve an average of one participant per month. Recruitment and screening data will be monitored at individual sites, and reasons for not meeting the targets, explored. Where applicable and if necessary, the need to substitute sites for those unable to meet the target will be considered.

### Qualitative sub-study

A qualitative study with a subset of trial participants (approx. 30–40) has also been incorporated. This nested study will aim to assess the acceptability and adherence to the treatment interventions in the trial. This approach will facilitate evaluation of the interventions based on the experiences of patients receiving the intervention and will be used to inform the results of the main trial.

The protocol conforms to the Standard Protocol Items: Recommendations for Interventional Trials (SPIRIT) guidelines [20]. The SPIRIT checklist is provided as Additional file 1. The data collected at each time point will be as described in Table 1.

**Table 1:** Summary of outcomes and assessment schedule

Timepoint	Visits				Follow-up – postal/e-mail questionnaire		
	Screening	Enrolment & Baseline	Intervention	Re-assessment^a^	6 months	12 months	18 months
**Informed consent**		X					
**Patient demographics**		X					
**Medical history**		X					
**Physical examination**				X^a^			
**MRI (as part of routine practice)**	X						
**Eligibility assessment**	X						
**Randomisation**		X					
**Adverse event reporting**^**⊥**^					x	X	X
**Treatment:** Operation/rehabilitation			X				
**Questionnaire**							
Knee Injury and Osteoarthritis Outcome Score (KOOS)		X			X^b^	X^b^	X
Return to activity/ level of sport participation – modified Tegner		X					X
Health economics – EQ. 5D		X			X	X	X
Complications					X	X	X
Knee-specific patient-reported outcomes, ACL-QOL		X			X^c^	X^c^	X
Patient satisfaction					X^d^	X^d^	X

Adverse events can be reported throughout follow-up (e.g., clinical events form, follow-up questionnaires) and in the final readmission check-list

^a^ Clinical assessment appointment for participants randomised to rehabilitation requiring reassessment due to continued problems with instability

^b^ Only KOOS4 (Pain, Symptoms, Function in sport and recreation and knee related Quality of life subscales)

^c^ ACL-QoL – only questions 1–5, 11 and 12

^d^ Questions about your treatment and your health – 1 question

### Study setting

A total of 320 patients will be recruited from approximately 30 NHS orthopaedic units, including district general and teaching hospitals, from across the United Kingdom over a period of 2 years. Application to the UK Clinical Research Network (UKCRN) will be made to help facilitate recruitment and support the study.

The sites will be selected on the basis of having an established practice of ACL reconstruction and an experienced ACL reconstruction knee surgeon and physiotherapy team capable of providing contemporary care. All orthopaedic surgeons involved in performing the surgical intervention of the study will be designated as having expertise in soft tissue knee surgery as indicated in the Best Practice for Primary Isolated Anterior Cruciate Ligament Reconstruction guidelines (BOA, [21]), with a minimum experience of 50 procedures in their career. Non-surgical management (rehabilitation) will be delivered (or closely overviewed) by senior physiotherapists (UK Health and Care Professions Council (HCPC) registered) with experience of ACL injury regimens.

Before a site is included, evidence of its patient throughput will be reviewed. In addition, the protocol will be discussed with the clinical team to ensure that it would be feasible to run the study at the site. As the time interval between referral and treatment can be variable in the current care pathway, the time period between randomisation and intervention will be standardised (as much as possible) within the study. Only sites that can offer treatment (ACL surgery or rehabilitation) within the 18 week pathway, (in line with current NHS waiting time targets) will be recruited. In addition, as part of the site selection process, documentary evidence of the use of a rehabilitation protocol that reflects the guidelines set will be required. Agreement to maintain consistency (adhere to the guidelines) with the aspects of the surgical intervention as laid out by the study protocol, will also be a requirement.

Regular contact and support will be maintained with study sites to help ensure that the protocol is carried out as planned.

### Participants

#### Eligibility of trial participants

Patients referred to any of the participating sites with symptomatic knee problems (instability) consistent with an anterior cruciate ligament injury will be assessed for eligibility by the principal investigator (PI) or a delegated clinical member of the research team. Anterior cruciate ligament deficiency (ACLD), either partial or complete tear, will be confirmed at the routine outpatient appointment through clinical assessment and MRI scan.

Anterior cruciate ligament tears can occur as isolated injuries but more commonly occur in conjunction with injuries to other structures of the knee, including menisci, articular cartilage and collateral ligaments. Apart from the pathology detailed in the exclusion criteria below, all other patients with an ACL tear combined with associated injuries can be considered for participation in the trial.

#### Inclusion Criteria

Participants can be included if they meet the following criteria:

- Participant is willing and able to give informed consent for participation in the study.
- The patient is male or female, aged 18 years or above.
- Symptomatic ACL deficiency of the native ligament* (instability-episodes of frank giving way or feeling unstable) with ACL deficiency (either partial or complete tear) is confirmed using clinical assessment and MRI scan.

* Patients who have undergone primary ACL reconstruction on the index knee are not eligible.

#### Exclusion criteria

The participant may not enter the study if any of the following apply:

- Patient is in the acute phase of primary ACL injury; that is, the patient has not recovered from any acute symptoms relating to their initial ACL injury*.
- Patient has had previous knee surgery (other than diagnostic arthroscopy or partial meniscectomy) to the index knee or concomitant severe injury to the contra-lateral knee.
- Patient has meniscal pathology with characteristics that indicate immediate surgery. i.e., locked knee or large bucket handle or complex cartilage tear producing mechanical symptoms.
- Patient has knee joint status of grade 3 or 4 on the Kellgren and Lawrence scale [22].
- Patient has grade 3 medial collateral ligament (MCL)/lateral collateral ligament (LCL) injury, associated posterior cruciate ligament (PCL)/posterolateral corner (PLC) injury.
- Patient has inflammatory arthropathy.
- Patient is pregnant. Any pregnancy will be determined before patient receives an MRI scan.

*Patients with pre-existing ACL deficiency presenting with acute symptoms (from a recent instability episode) can be considered for inclusion.

### Interventions

The study compares two routine and well-established management strategies for patients with symptomatic non-acute ACL deficient knees: a) non-surgical management (rehabilitation) and b) surgical management (reconstruction).

Both interventions are routine NHS treatments. Intervention content is based on a minimal set of pre-established criteria in order to ensure the integrity of the comparison while allowing for varying in practice in delivering the interventions between both surgeons and physiotherapists (see below). This pragmatic approach to the delivery of the intervention will allow the management approach to reflect current practice and outpatient resources within the NHS thus aiding generalisation, yet include minimal levels of standardised quality and content for both interventions.

The description and standardisation of the interventions for the trial has been informed from several sources. These include an overview of the best evidence to date, the results of a survey of ACL surgeons, synthesis of current practice guidelines/rehabilitation protocols from UK Trusts and consensus meetings (surgeon/physiotherapist).

Operations will be carried out according to the discretion of the participating surgeon. Two types of ACL reconstruction are commonplace and acceptable: one using a patella tendon graft and the other using a hamstring graft. The rehabilitation content for the conservative arm will be based on standard care for the participating site. Minimal levels of quality and content have been set for both interventions (see below).

- Non-surgical management (rehabilitation)

Patients randomised to rehabilitation will be referred to their nearest physiotherapy department and undergo non-surgical management (rehabilitation) delivered (or closely overviewed) by a senior physiotherapist with experience of ACL injury regimens. The routine rehabilitation protocol used at the participating site will be followed.

As part of the site selection process, documentary evidence of the use of or willingness to adopt a rehabilitation protocol that reflects the guidelines of the mandatory aims/goals set for the study rehabilitation intervention (see below) will be required. Part of the requirement will be for the site to be in a position to provide a minimum of six rehabilitation sessions delivered over at least a 3-month period.

The rehabilitation protocol will include the following components:

- Evidence of interventions aimed at achieving the mandatory aims/goals:

1. Control of pain and swelling
2. Regaining range of movement
3. Improving neuromuscular control
4. Regaining muscle strength
5. Achieving normal gait pattern
6. Returning to function/activity/sport

- Clearly identified progression milestones.
- Return to sport criteria.
- Identification criteria for poor or non-progression.

Rehabilitation protocols commonly used in clinical practice consist of a progressive programme [23], designed to rebuild muscle strength, re-establish joint mobility and neuromuscular control, and enable patients to decrease the risk of re-injury and return to previous levels of activity [24].

As little consensus exists in the literature over the most effective rehabilitation protocol [25], variation in the specific exercises carried out and the use of adjuncts (such as cryotherapy) to reach these aims is permitted. Examples of exercises used to reach the aims will be documented in a physiotherapy case report form (PCRF). Flexibility is permitted to adapt treatment to individual needs with no timelines specified for progression. Evidence of individual progression, however, will be documented in the PCRF. A physiotherapy case report form (PCRF) will be used to facilitate recording of the rehabilitation interventions to monitor for fidelity to these guidelines.

The progress of patients who have been randomised to non-surgical management (rehabilitation) will be monitored by their treating physiotherapist. If, after a minimum period of at least 3 months of rehabilitation (or before, if instability or symptoms are deemed substantial), the participant continues to experience symptomatic knee instability and/or symptoms related to associated pathology, i.e., pain or locking, the suggestion is that the non-surgical management has failed. If the patient meets the criteria listed below, a review appointment with the surgeon will be arranged. If following surgical assessment, a decision is made to proceed with ACL reconstruction surgery, the participant will be listed for surgery, as per usual practice.

All other clinical follow up will occur as per routine practice at each participating site. The criteria for change in status (from non-surgical to surgical intervention) after a minimum of 3 months of rehabilitation were confirmed at a consensus meeting (surgeon/physiotherapist) 20 January 2016. The consensus group agreed that 3 months is considered the minimal time needed for the rehabilitation to provide any effect. The criteria for surgery include one or more of the following:

- Continued feeling of knee instability and/or symptoms, i.e., pain or locking, related to the associated pathology
- At least two episodes of physical giving way of the knee
- Unable to return to a Tegner activity level 2 points below pre-injury status

Outside early conversions (inside 3 months), the above criteria assume all patients will have undergone a comprehensive rehabilitation regime according to the study protocol – the physiotherapy case report form will provide evidence of completion and fidelity.

The operation case report form (OCRF), detailed below, will be used to document the operation and monitor compliance with the intervention guidelines. Post-operative rehabilitation will also be recorded for this group.

b) Surgical Management (Reconstruction)

Patients randomised to reconstructive surgery will be placed on a waiting list to undergo a standard ACL reconstruction procedure (ACLR). All surgical reconstructions will be patella tendon or hamstring tendon, depending on the surgeon’s preference. Any physiotherapy advice and any treatment aimed at the acute presentation (i.e., swelling, regaining range of motion, etc.) prior to surgery can be given, but no formal ACL rehabilitation programme or specific ACL remedial exercise prescription beyond basic maintenance exercises. All other care will be routine, including immediate post-operative care.

For the purpose of this pragmatic trial, the surgical ACL reconstruction will be performed according to standard local policy, provided the minimal quality and care components as described below are consistent.

All patients will undergo pre-operative evaluation to assess their clinical condition and co-morbidities. During the trial, the operative intervention will take place adhering to their local trust policies for anaesthesia, DVT prophylaxis and antibiotic use.

Surgery will be performed or supervised in theatre by a specialised consultant knee surgeon with recognised expertise in ACLR (will have performed at least 50 previous ACL reconstructions). Patients will be placed supine on the operating table and set up for an arthroscopic knee procedure. Tourniquet use will be applied as per local protocols. The incisions (commonly anteromedial and anterolateral) will be placed at the surgeon’s discretion. Under arthroscopic guidance, any remaining remnants of the torn ligament will be removed, and anatomical landmarks within the knee, identified. The desired graft will be harvested from a separate incision. Using a combination of direct vision and instrumentation, tibial and femoral tunnels will be placed to receive the graft. The graft will be introduced into the pre-prepared tunnels and once it is in situ and under tension, the graft will be fixed in position. Any additional surgery to other structures in the knee, e.g., menisci, will be conducted as per routine practice. All incisions will be sutured and bandaged, as per local protocols.

Patients will be engaged in a post-operative rehabilitation programme as per standard care at the participating hospital. Note the initial content of post-operative physiotherapy is different from that for non-surgical management in that some aspects of graft protection and caution are necessary following ACL reconstruction.

An operation case report form (OCRF) will be used to document the operation and monitor compliance with the intervention guidelines. The content of and attendance (adherence) to the post-operative rehabilitation will also be recorded for this group.

No rapid changes are expected in the content or delivery of either of these management approaches in the near future.

### Outcomes

The primary outcome for the study is the Knee Injury and Osteoarthritis Outcome Score (KOOS4) at 18 months post randomisation. This outcome measure is derived from four of five subscales: pain, symptoms, difficulty in sports and recreational activities and knee-related quality of life [26, 27], with scores ranging from 0 to 100, and a higher score indicating better health. KOOS is a validated patient-reported outcome used in ACL research (including recent RCT of acute ACL patients [26, 28] and large scale databases, i.e., the National Ligament Registry [29, 30]). The KOOS4 is sensitive and specific for detecting functional deficits due to knee instability.

The secondary outcomes are as follows:

#### Return to activity/level of sport participation

The return to activity/level of sports participation is measured by the Modified Tegner [31]. The activity level assessed using the Tegner scale is graded from 1 (low activity levels) to 10 (professional level).

#### Intervention-related complications

Any complications associated with undergoing ACL deficiency treatment will be recorded. This includes the following for the surgery group: re-admission, delayed hospital discharge, infection, unexpected poor range of movement (stiffness), excess bleeding, continued swelling, episodes of giving way, and a continued feeling of instability. For the non-surgical group, in includes continued swelling and episodes of giving way.

#### Generic quality of life

The EuroQol EQ-5D-5 L is a validated, generic, self-reported outcome measure covering five health domains that are used to facilitate the calculation of quality-adjusted life years (QALYs) in health economic evaluations. The original EQ-5D questionnaire contained three response options within each of five health domains (mobility, self-care, usual activities, pain/discomfort and anxiety/depression) [32]). More recently, the EQ-5D-5 L has been developed to overcome problems with ceiling effects and to improve sensitivity [33]. The 5 L version consists of the same five domains as the original but with five response options.

#### Knee-specific patient-reported outcomes

All five subscales of the KOOS [27] will be included (the fifth scale being activities of daily living).

#### Anterior Cruciate Ligament Quality of Life Score (ACL-QOL)

This score, as described in [34] is a validated 32-item, knee-specific measure for chronic ACL deficiency, divided into five sub-scales which include symptoms and physical complaints, work-related concerns, physical activity and sports participation, lifestyle issues and social and emotional concerns. The overall score is calculated (0–100), with higher scores indicating better outcome.

#### Resource usage data

Detailed resource use data on initial treatments received (surgical reconstruction or rehabilitation) and on subsequent healthcare contacts including re-operations (surgery arm), subsequent surgical reconstructions (rehabilitation arm), surgery-related complications, further rehabilitation, and primary care and other secondary care contacts out to 18 months post-randomisation are secondary outcomes. In addition, data will be collected on the ability to work (e.g., sickness absences/return to work number of days off work and subjective working ability).

#### Expectations of return to activity and confidence in relation to the knee

Patients will be questioned on their expected outcome in relation to their return to activity and on how confident they feel about doing so, considering any limitation related to their injured knee. This will be assessed by the Anterior Cruciate Ligament Quality of Life Score (ACL-QOL) [34].

#### Patient satisfaction

A simple Likert scale will be used to assess satisfaction with the outcome of treatment.

The outcomes reflect consensus opinion and the reference standard for evaluating ACL injury/reconstruction [35]. A specially convened PPI focus group indicated that the KOOS score, despite being the most valid tool available and having been used in most major ACL studies, did not reflect the entire scope of symptoms for ACLD patients.

The schedule for the baseline and follow-up assessments is shown in Table 1.

### Participant timeline

**Fig. 1:**
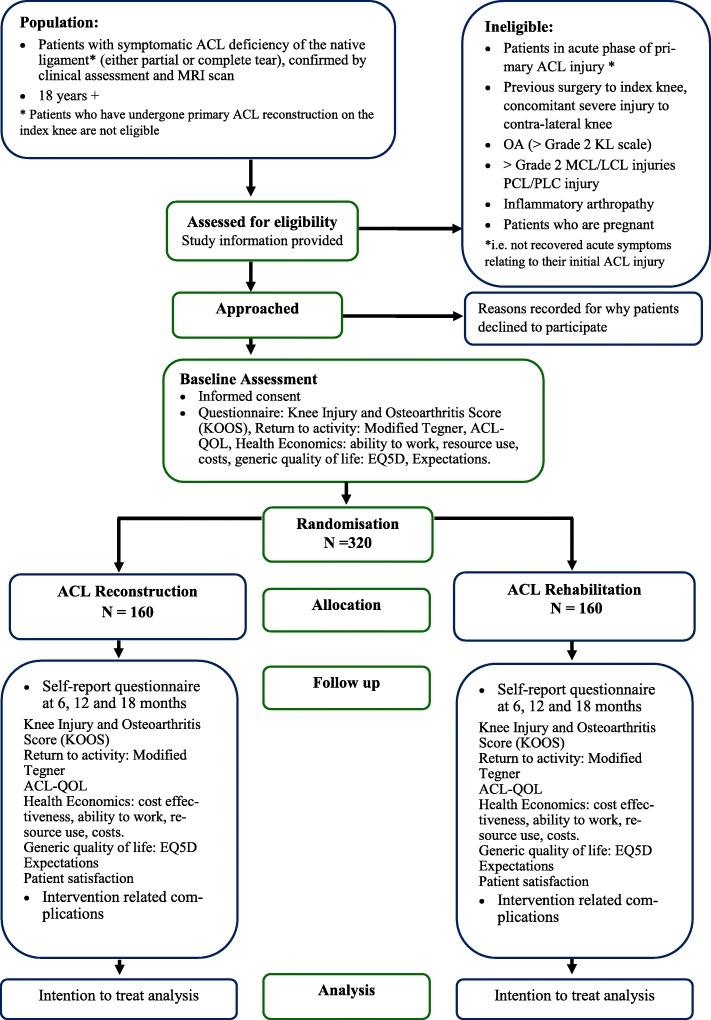
Participant flow diagram ACL SNNAP trial (Details of specific outcomes collected at each of the follow-up time points are outlined in Table 1)

Patients referred to any of the participating sites with symptomatic knee problems consistent with an anterior cruciate ligament injury will be assessed for eligibility by the PI or a delegated clinical member of the research team. The process of patient identification and recruitment will vary depending on the local treatment pathways at each participating site. The flowchart in Fig. 1 details the recruitment process.

As per routine practice anterior cruciate ligament deficiency (ACLD), either partial or complete tear, will be confirmed at an outpatient appointment through clinical assessment and MRI scan.

Anterior cruciate ligament tears can occur as isolated injuries but more commonly occur in conjunction with injuries to other structures of the knee, including the menisci, articular cartilage and collateral ligaments. Apart from the pathology detailed in the exclusion criteria above, i.e., grade 3 MCL/LCL, associated PCL/PLC and meniscal pathology considered significant enough for immediate repair/resection, all other patients with an ACL tear combined with associated injuries can be considered for participation in the trial.

The participating surgeon or member of the clinical team will initially approach potential participants who meet the eligibility criteria and inform them of the study. Patients who express a potential interest in participating would then be referred to a research nurse/physiotherapist for further details about the study and written information. Patients who wish to participate will complete an informed consent form and baseline questionnaire. If a patient would like more time to consider participation, the research team will agree an arrangement with the patient to confirm their decision.

Situations may arise where a MRI scan is requested by the clinician prior to the confirmation of ACL deficiency. In these cases, information can be provided to the patient to inform them of the study, and possible participation can be discussed once the diagnosis is confirmed.

The baseline questionnaire will include the following outcome measures: KOOS, EQ-5D-5 L, Modified Tegner and ACL-QOL, as detailed in the outcomes section above. Details of the baseline level of ACL injury and associated knee pathology from the MRI report will also be recorded.

### Screening and eligibility assessment

Screening logs will be implemented at each of the recruiting sites in order to document the reasons for non-inclusion in the study (e.g., reason they were ineligible, or declined to participate). The central study office will use this to monitor recruitment at sites and to inform the CONSORT diagram.

### Informed consent

The patient must personally sign and date the latest approved version of the Informed Consent form before any study specific procedures are performed.

Written and verbal versions of the Participant Information and Informed Consent will be presented to the participants detailing no less than the following: the exact nature of the study, what it will involve for the participant, the implications and constraints of the protocol, the known side effects and any risks involved in taking part. As will be clearly stated, the participant is free to withdraw from the study at any time for any reason without prejudice to future care, without affecting their legal rights, and with no obligation to give the reason for withdrawal.

The participant will be allowed as much time as wished to consider the information, and the opportunity to question the investigator, their GP or other independent parties to decide whether they will participate in the study. Written informed consent will then be obtained by means of participant dated signature and dated signature of the person who presented and obtained the informed consent. The person who obtained the consent must be suitably qualified and experienced, and must have been authorised to obtain the consent by the local site’s principal investigator. A copy of the signed informed consent will be given to the participant. The original signed form will be retained at the study site, a copy will be placed in the patients’ medical notes, and another copy will be sent to the study co-ordinating team at Oxford for storage for central monitoring purposes.

The qualitative interviews will take place in a selected number of sites and with a small sample of participants. A separate information sheet and consent form will be used for the qualitative study. Written informed consent will be taken before the interview. A copy of the signed informed consent will be given to the participant. The original signed form will be retained at the study office in Oxford.

### Assignment of interventions

#### Sequence generation

Following consent, once trial eligibility has been confirmed, participants will be randomised into the trial by a member of the local research team. Randomisation will be performed using a web-based automated system provided by Fr3dom limited. The allocation will be generated using permuted block randomisation with varying block sizes stratified by baseline KOOS score and site. Patients will be allocated a study number and randomised on a 1:1 basis to receive one of two management options: non-surgical management (rehabilitation) or surgery (reconstruction).

#### Allocation concealment mechanism and implementation

The centrally managed randomisation will ensure allocation concealment and prevent selection bias. Following randomisation, the allocation details will be displayed on the web-based system for each participant, and an automated e-mail will also be sent to the designated member of the research team at the site to inform them of the allocation. A standard letter will be used to inform the admissions, care pathway co-ordinators, and GP (with patient consent) of allocation.

#### Blinding (masking)

Due to the nature of the interventions, neither participants nor healthcare practitioners (surgeons and physiotherapists) can be blinded to receipt of the intervention.

#### Follow-up

Follow up for study purposes will be by patient self-reported questionnaire completed using a web-based data collection system. The option of being able to fill out the follow-up questionnaires in a hard copy and returning via post will also be available. Non-response will be minimised through use of multiple reminders such as web-based messages, phone calls and texts.

The 18-month (primary endpoint) follow-up questionnaire will contain the following outcome measures: KOOS, EQ-5D-5 L, Modified Tegner, ACL-QOL, and patient satisfaction and will be sent out at 18 months post randomisation to all participants (as detailed above). A shortened version of the follow-up questionnaire is sent out at the 6- and 12-month time points. Details of the specific outcomes collected at each of the follow-up time points are outlined in Table 1. The questionnaires will also ask participants if they have returned to see a healthcare professional or been admitted to hospital in relation to complications with their study knee. The trial manager in Oxford will follow up any complications reported by participants with the research team at the participant’s local hospital. Further details about the event will be collected and recorded on a complications form. Participants randomised to rehabilitation will be referred to their nearest physiotherapy department and undergo standardised rehabilitation (to acceptable practice) delivered by physiotherapists with experience of ACL rehabilitation (as described above). A physiotherapy case report form will be used to facilitate recording of the rehabilitation intervention and used to monitor compliance with the mandatory aims/goals of the rehabilitation intervention.

Participants randomised to reconstruction will be placed on a waiting list to undergo ACL surgery. An operation case report form (OCRF) will be used to document the operation and monitor compliance with the intervention guidelines. As a period of post-operative rehabilitation is part of the standard treatment following ACL reconstruction, attendance (adherence) to rehabilitation and content will also be recorded for this group. The schedule of enrolment and assessments is shown in Table 1.

#### Data collection methods

Data collection will be facilitated by a custom designed database created by Fr3dom, using the Fr3PROMS proprietary platform. A guide explaining how to use the ACL SNNAP electronic data collection system will be provided to every site.

Data from the web-based questionnaires will be captured automatically after the participant completes the online questionnaire. Data from any paper questionnaires will be entered manually into an electronic database by the local study team at participating sites or by the central study office in Oxford.

All electronic data will be captured via an xml schematic, encrypted and written down securely once a survey is saved on the device. Data is encrypted and stored on the device. Transfer happens securely over mobile, Wi-Fi, or wired connection. The encrypted data is sent via a secured connection to the secure data centre. Industry standard protocols and processes are used to ensure the highest secure environment.

Access to data from the client is through an intelligent SSL fire wall and can only be accessed by authorised users.

A study specific participant number and/or code in any database will be used to identify the participants. Any patient related data transferred between the main study office and participating sites will be identifiable only with the patient’s unique study number. If more identifiable information is required, secure measures such as registered post, courier, or nhs.net email accounts will be utilised. For quality control reasons, the main study team may initiate monitoring of site files and data collection forms.

The chief investigator will act as data custodian for the trial. Direct access will be granted to authorised representatives from the sponsor and host institution for monitoring and/or audit of the study to ensure compliance with regulations.

#### Qualitative sub-study

In addition to the main study, a qualitative sub-study will also be conducted. This nested study aims to assess the acceptability and adherence to the treatment interventions in the trial. This will facilitate evaluation of the interventions based on the experiences of the patients receiving the intervention and will be used to inform the results of the main trial.

After receiving the intervention, a small number of participants (approx. 30–40) who consented at trial entry to being contacted about the interview study will be invited to participate in semi-structured interviews.

A separate information sheet and consent form will be used for the qualitative interviews. Interviews will be held at a convenient time and location for each participant. Ideally interviews will be undertaken face-to-face; however, given the geographical spread of participants, performing some interviews by telephone or online (e.g., Skype) may be more practical. Participants may choose to have the interview within their own home, in which case the researcher must adhere to the Oxford University and/or Trust lone worker policy. Previous experience suggests that each interview will last between 30 and 45 mins.

Purposive sampling will be carried out to achieve a sample that includes participants who were randomised to the surgical or rehabilitation intervention and those in the rehabilitation arm who subsequently decide to have surgery.

All interviews will be audio recorded, transcribed verbatim and analysed with the assistance of Nvivo qualitative data analysis software (QSR International Ltd., Melbourne, Australia). Field notes and memos will be recorded using a digital notepad. Participants will be offered the opportunity to check their transcript, providing them an opportunity to remove anything with which they do not feel comfortable.

### Sample size

#### The number of participants

In total, 320 participants will be recruited to the study. The minimal clinically important change (MIC) for the KOOS score is 8–10 points [36]. Estimates of the minimal detectable change (MDC) for the two KOOS subscales most relevant for ACLD vary between five and 12 points (Symptoms 5–9, and Sport/Rec 6–12) [36]. A mean target difference of eight points in the primary outcome, KOOS4, along with a standard deviation of 19 (the highest value observed in a trial of acute patients at baseline amongst the KOOS subscales) were assumed [37, 38]. Given these assumptions, 120 participants per group are required (1:1 allocation, 240 in total) to achieve 90% power at two-sided 5% significance level in the absence of any clustering of outcome. However, to ensure sufficient power, clustering (clsampsi Stata command [39]) has been allowed for by conservatively assuming an intra-cluster correlation (ICC) of 0.06 [40] and cluster size n, mean (SD) of 26, 5 (12) and 43, 3 (5) for the ACL reconstruction and rehabilitation groups, respectively. This leads to the larger number of 130 participants per group (260), which has just over 80% power. Given the conservative nature of the assumed values and the anticipated gain in precision from adjusting for the baseline scores and other randomisation factors, actual power is likely to be higher even in the presence of clustering. To allow for just over 15% missing data (response in a similar trial [26]), 320 participants will be needed.

An interim analysis will be carried out to estimate the magnitude of clustering for the 6 months KOOS4 outcome once data is available for 100 participants. Based upon this, a decision as to whether the sample size should be increased to allow for a greater level of clustering than anticipated will be made.

### Statistical methods

A single main analysis will be performed at the end of the trial once the 18-months of follow-up data are available. All principal analyses will be based on the intention-to-treat principle, analysing participants in the groups to which they are randomised. A secondary analysis of the primary outcome will explore the impact of non-compliance to treatment allocation. Statistical analyses will be pre-specified in a statistical analysis plan, which will be agreed by the Trial Steering Committee, prior to conducting the statistical analysis.

An independent data monitoring committee (DMC) will meet early in the course of the trial to agree its terms of reference and will review confidential interim analyses of accumulating data (including the interim analysis of clustering).

### Statistical analysis

#### Analysis of outcome measures

The primary outcome measure (KOOS4 overall score) will be compared using a regression model with adjustment for the randomisation variables. Secondary outcomes will be analysed using generalised linear regression models with adjustment for randomisation and baseline variables, as appropriate. Statistical significance will be at the two-sided 5% level, with corresponding confidence intervals derived, and the analyses will be carried out in Stata [41]. Exploratory subgroup analyses will explore the possible treatment effect modification of clinically important baseline factors (age, gender, high versus moderate or light physical activity as measured by the modified Tegner score, and the KOOS4 overall score), through the use of a treatment by factor interaction and will be interpreted cautiously. The impact of missing data and non-compliance will be explored by utilising multiple imputation and complier average causal effect (CACE) approaches, respectively. Clustering will be quantified as the ICC with associated 95% confidence interval, which will be calculated using a bootstrapping approach.

#### Cost-utility analysis

A health economic evaluation (more specifically a cost-utility analysis) will be designed as an integral part of the ACL SNNAP Trial and will be conducted from NHS and societal perspectives. Detailed resource use data will be collected for each trial participant on initial treatments received (surgical reconstruction or rehabilitation) and on subsequent healthcare contacts including re-operations (surgery arm), subsequent surgical reconstructions (non-surgical rehabilitation arm), surgery-related complications, further rehabilitation, and primary care and other secondary care contacts out to 18 months post-randomisation. Beyond the healthcare sector, data will also be collected from each patient about their contacts with private healthcare practitioners, unpaid informal care provided by relatives and/or friends, and time away from paid employment. Resource use data will be costed using national average unit costs from a variety of established sources [42–44].

Patients will complete the EuroQol EQ-5D-5 L questionnaire at baseline, 6 months, 12 months, and 18 months, and responses will be converted into single index scores [33]. A new value set is being developed that will allow England to derive scores from the EQ-5D-5 L, but this set is not expected to be available before data analysis begins [45]. In the absence of the new value set, the scoring of the EQ-5D-5 L description system will use the mapping algorithm by Van Hout and colleagues, as recommended by NICE [46, 47]. These scores will be used to calculate quality-adjusted life years (QALYs) for each trial patient out to 18 months post-randomisation.

Resource use, costs, and QALYs to 18 months will be summarised using means and standard deviations for each trial arm. Mean differences and 95% confidence intervals for differences will be used when comparing data between trial arms. Incremental analyses will be performed, and if appropriate, the incremental cost-effectiveness ratio (ICER) will be used to express results in terms of an additional cost per QALY gained. Sampling uncertainty around the ICER will be explored using non-parametric bootstrapping. Parameter uncertainty will be examined using sensitivity analysis. Cost-effectiveness acceptability curves will be used to estimate the probability of the interventions being cost-effective at a maximum willingness to pay of £20,000 to £30,000 per QALY gained.

Potential longer-term cost-effectiveness will be estimated by extrapolating costs and health outcomes beyond the time horizon of 18 months. Extrapolations will be based on a modelling of the rates of re-operations observed in the surgery arm of the trial and subsequent surgeries observed in the rehabilitation arm of the trial.

### Data monitoring

Details of the committee personnel can be found in the Acknowledgments section.

#### Trial management group

The trial will be managed through the Surgical Intervention Trial Unit (SITU) and OCTRU, University of Oxford, and the research team’s trial management group (TMG). The TMG will include the chief investigator, lead collaborative investigators and trial staff. The principal investigators at the recruiting sites are responsible for the study conduct at their sites. SITU will provide day-to-day support for the sites and provide training through investigator and research practitioners meetings, site initiation, phone calls and routine monitoring.

The study will be conducted according to the principles of GCP. A risk assessment will be conducted before the trial starts and a proportionate monitoring plan will be drawn up and used for the trial.

#### Trial steering committee

A trial steering committee with an independent chair will provide overall supervision of the trial. The TSC will meet every 6 months or more/less frequently if circumstances dictate during the trial. Its role is to monitor progress and supervise the trial to ensure it is conducted to high standards in accordance with the protocol, the principles of GCP, relevant regulations and guidelines with regard to participant safety.

#### Data safety monitoring committee

A data monitoring committee (DMC) will be convened with independent statistician, clinician and chairperson to provide independent review. Its purpose is to monitor efficacy, safety and compliance data. The DMC will have access to unblinded study data. During the recruitment period, interim analysis will be supplied, in the strictest confidence, together with any other analyses that the committee may request.

### Discontinuation and withdrawal of participants

Each participant has the right to withdraw from the study at any time. In addition, the Investigator may discontinue a participant from the study at any time if the investigator considers it necessary for any reason including:

- Ineligibility (either arising during the study or retrospectively having been overlooked at screening)
- Significant protocol deviation
- Significant non-compliance with treatment regimen or study requirements
- Withdrawal of consent
- Loss to follow up

Participants will remain in the study unless they chose to withdraw consent or if they are unable to continue for a clinical reason. The reason for withdrawal will be recorded on the study change of status form. All other changes in status with the exception of formal withdrawal of consent will mean the participant is still followed up for all study outcomes wherever possible.

### Adverse event reporting

Adverse event reporting will be undertaken in accordance with the National Research Ethics Services (NRES) guidelines, Research Governance Framework and OCTRU Standard Operational Procedure guidelines for non-CTIMP studies.

The study involves routine ACL reconstruction surgery and rehabilitation for the management of symptomatic ACL deficiency. No additional risks to patients exist. The participants will either undergo ACL reconstruction or rehabilitation as per standard management. Patients will be informed of the standard risks associated with anaesthetic and ACL reconstruction operations.

Possible (expected) complications and consequences are as follows:

All ACL reconstruction procedures, whether primary surgery or revision, carry a risk of anaesthesia-related problems, which can include death; morbidity, including wound infection; bleeding, intra- and post-operatively; PE; DVT; confirmed CVA; confirmed MI; and complications secondary to existing co-morbidity, e.g., ischaemic heart disease, septicaemia, the need for blood transfusion and revision operation.

Specific complications following ACL reconstruction procedures include patella fracture, patella tendon avulsion, anterior knee pain, vascular injury and bleeding, femoral tunnel blowout, nerve damage (including numbness or weakness), complex regional pain, lack of extension/fixed flexion deformity, stiffness, infection, graft failure and continued instability, delayed wound healing, continued or worsened pain, fracture, compartment syndrome, swelling, contralateral graft harvest and newly acquired meniscal pathology.

Specific complications following rehabilitation include continued instability and subsequent newly acquired meniscal pathology and pain. These complications may result in the need for further surgery. Details of all complications will be collected and recorded as detailed on a clinical events form. A final readmission checklist will be undertaken by the research staff on hospital records at 18 months post-randomisation to ensure that all complications data is collected from all participants (i.e., those who had not returned a questionnaire). Data from any readmission events identified will be recorded on the clinical events forms.

A serious adverse event for ACL SNNAP is defined as any untoward medical occurrence that was both unexpected and related to the study treatments that 1) resulted in death, 2) was life threatening, 3) required inpatient hospitalisation or prolongation of existing hospitalisation or 4) resulted in persistent or significant disability/incapacity.

If a SAE form is completed detailing any possible related and unexpected SAEs, the chief investigator (CI) or delegate will be notified. If, in the opinion of the local PI and CI, the event is confirmed as being related (resulted from administration of any of the research procedures) and unexpected (i.e., not listed as a possible expected occurrence detailed above), the CI will submit a report to the main REC and the study sponsors within 15 days of the CI becoming aware of it.

### Definition of end of study

The end of study is the date when all analysis is completed, and the monograph is submitted to the funding body.

### Dissemination policy

The trial investigators will be involved in reviewing drafts of the manuscripts, abstracts, press releases and any other publications arising from the study. Authors will acknowledge that the study was funded by National Institute for Health Research (NIHR) (Health Technology assessment Programme (project reference: 14/140/63)). Authorship will be determined in accordance with the International Committee of Medical Journal Editors (ICMJE) guidelines, and other contributors will be acknowledged.

We plan for the findings to be published in a high impact peer-reviewed journal, which will allow for the results to be disseminated across the orthopaedic and rehabilitation communities, the wider medical community and NICE. In addition, we plan for the findings of the study to be presented at several conferences such as, the annual meeting of the British Association of Knee Surgery (BASK), British Orthopaedic Association (BOA) and the Physiotherapy Research Society (PRS). We anticipate the findings will be used to develop evidence-based guidance to inform the management of patients with non-acute ACL deficiency.

## Discussion

The ACL SNNAP trial will aim to address the gap in the evidence base regarding the clinical and cost effectiveness of two routine management approaches, non-surgical (rehabilitation) and surgical (reconstruction), and inform standards of care for patients with non-acute ACL deficiency. Conducting trials that include non-surgical and surgical intervention comparators, however, can be challenging. Factors such as crossover between intervention groups, treatment preferences of patients and clinicians, if not adequately addressed, may introduce significant bias and threaten the viability of the trial, and also the validity of the results. Several aspects therefore warrant further discussion in relation to this trial.

### Threats to recruitment

The treatment preference of the population under investigation and the equipoise and preference of the surgeon delivering the intervention are considered as potential threats to recruitment and the successful completion of this study.

Eligible patients for the study will present with non-acute ACL deficiency; that is, they have recovered from any acute symptoms relating to their initial ACL injury. Depending on time since injury, some patients will feel they have already attempted conservative treatment during this period. These patients, who have the potential to be allocated to continued non-surgical treatment, are likely to have a stronger preference for surgery.

On the clinical side, recruiting surgeons are often the primary management decision makers for this population (sometimes with input from therapists). Some issues of bias and preference for surgery may occur in the recruitment process. The internal pilot will evaluate the safeguards established to counteract this potential bias.

### Compliance to treatment

Compared with trials of similar procedures (for example, two surgical procedures), non-compliance between intervention groups can potentially result in an imbalance between treatment arms in trials comparing non-surgical and surgical procedures, as “crossover” can only occur in one direction (towards surgery). This potential imbalance could result in complexities with interpretation of the results in these types of trials. ACL SNNAP was designed as a management trial with the option for later ACL reconstruction included as part of the Rehabilitation arm, frequent change in status to surgical management from non-surgical management therefore is not considered a threat to study completion or a problem for analysis. The need for surgery, based on the firm standardised criteria outlined in the protocol, would indicate failed management and will be an outcome measure in itself. Obviously, patients will not be able to change from surgery to conservative management once surgery has been performed, in that the operation cannot be undone. However, some patients may wish to change status immediately after randomisation after finding themselves allocated to a (self-perceived) inferior management group. Lessons learnt from previous qualitative work (CSAW study, Arthritis Research UK) [48] will be utilised to avoid this.

### Loss to follow-up

Data collection for this population—the ACL injury group tends to be younger (18–40 years old) and therefore very geographically mobile—can also be potentially challenging. Follow-up is by self-report questionnaire only at 6, 12 and 18 months and therefore not too onerous for patients. The internal pilot will check that such patients can be followed up consistently, and any innovative methods used to follow up a young mobile population are successful, i.e., web-based questionnaires, phone and text.

### Trial status

The current protocol is Version 4, dated 6th September, 2018. Recruitment to the ACL SNNAP trial commenced in March, 2017 and is ongoing at the time of manuscript submission. To date, 306 patients have been randomised (January 2020). Recruitment to the study is expected to finish by April, 2020. Details of the trial sites can be found on the trial website: https://snnap.octru.ox.ac.uk/

## Supplementary information

**Additional file 1.** SPIRIT 2013 Checklist: Recommended items to address in a clinical trial protocol and related documents.

## Data Availability

Study-related documents, e.g., patient information sheet, protocol are available on the NIHR website: https://www.fundingawards.nihr.ac.uk/award/14/140/63, or from the corresponding author on request.

## Abbreviations

ACL: anterior cruciate ligament
ACLD: anterior cruciate ligament deficiency
ACL SNNAP: Anterior Cruciate Ligament Surgery Necessity in Non-acute Patients
ACL-QOL: Anterior Cruciate Ligament Quality of Life Score
CONSORT: Consolidated Standards of Reporting Trials
EQ-5D-5 L: EuroQol-5 Dimensions, 5 level version
HTA: Health Technology Assessment
KOOS: Knee Injury and Osteoarthritis Outcome Score
LCL: lateral collateral ligament
MCL: medial collateral ligament
MRI: magnetic resonance imaging
PCL: posterior cruciate ligament
PLC: posterolateral corner
